# Viewing ourselves as nature: Holobiont literacy influences nature connectedness

**DOI:** 10.1007/s13280-026-02414-x

**Published:** 2026-05-06

**Authors:** Jake M. Robinson, Kate Robinson, Alexia Barrable

**Affiliations:** 1 Aerobiome Innovation and Research Hub (AIR Hub), College of Science and Engineering, Flinders University, Bedford Park, SA 5042 Australia; 2 College of Science and Engineering, Flinders University, Bedford Park, SA 5042 Australia; 3 Psychology, Sociology and Education Division, Queen Margaret University Drive, Musselburgh, EH21 6UU UK

**Keywords:** Holobiont, Microbiome, Nature connectedness, Nature connection, Nature relatedness

## Abstract

The human holobiont concept—humans as symbiotic assemblages of a host and trillions of microbes—offers a compelling lens for understanding human–nature relationships. This study examined whether: (a) prior holobiont knowledge correlates with nature connectedness, (b) exposure to holobiont information influences nature connectedness and (c) people feel more or less connected to microbes than to other natural entities. Using a randomised, blinded online survey (*n* = 190), participants were assigned to a holobiont treatment group (*n* = 91) receiving multimedia information or a control group (*n* = 99) receiving neutral content. Nature connectedness was measured before and after exposure. Results showed that prior holobiont knowledge was associated with higher nature connectedness, and, strikingly, that exposure to holobiont information significantly increased nature connectedness scores. No differences were found across nature types. These findings suggest that framing humans as holobionts may strengthen psychological connections to nature, with implications for environmental psychology, education and well-being.

## Introduction

The way humans conceptualise their relationship with nature has profound implications for environmental attitudes, behaviours and well-being. In some modern societies, nature has been perceived as something external to humanity—a separate entity to be observed, conserved or exploited (Beery et al. 2023). However, traditional ecological knowledge and emerging scientific insights challenge this dualistic perspective by reinforcing the reality that humans are components of ecosystems, deeply embedded within the natural world (Whyte 2013; Mistry and Berardi 2016; Barrable et al. 2024a, b). One particularly transformative “modern” concept in this regard is the *holobiont*, which reframes humans not as singular organisms, but as complex biological collectives composed of a host and its trillions of symbiotic microbes (e.g. bacteria, fungi, archaea, viruses, protists) (Van De Guchte et al. 2018; Robinson and Cameron 2020). For instance, “humans” are composed of approximately 47% somatic and 53% microbial cells (Sender et al. 2016). Moreover, the microbial genes in our bodies outnumber our “human” genes by 150 to 1 (Zhu et al. 2010). This has led some to suggest that, biologically speaking, we are “more microbial than human” (Parke 2021).

Understanding oneself as a holobiont—a multispecies assemblage—may alter perceptions of human–nature connectedness, reinforcing the reality that humans are embedded within and dependent on broader ecological networks. However, there is a dearth in microbiology literacy (Timmis et al. 2019), which may be contributing to “germophobia” or a fear of microbes, “dirt” and nature more widely (Robinson et al. 2021). This can have profound implications for human health (e.g. we need to be exposed to diverse microbial assemblages to train and regulate our immune system) and biodiversity (e.g. we need to feel connected to the rest of nature to enhance our desire to protect it) (Rook 2013). This study examines whether holobiont literacy, i.e. awareness and understanding of the holobiont concept, influences nature connectedness and whether exposure to holobiont-related information can enhance an individual’s sense of belonging to the natural world.

Nature connectedness refers to an individual’s affective, cognitive and experiential relationship with nature, or more broadly, one’s “relationship with the rest of nature” (Barrable 2019; Barragan-Jason et al. 2022). Higher levels of nature connectedness are associated with a range of positive outcomes, including increased pro-environmental behaviour (Mackay and Schmitt 2019) and greater psychological well-being in adults (Barragan-Jason et al. 2023) and in children (Barrable et al. 2024a, b). Research has shown that direct exposure to nature, as well as cognitive and educational interventions, can strengthen nature connectedness (Barrable et al. 2021; Pirchio et al. 2021). A meta-analysis of interventions has shown that both direct (e.g. visits, forest walks) and indirect (e.g. video or virtual nature) experiences of nature can enhance nature connectedness in adults (Sheffield et al. 2022). However, relatively little is known about how microbial literacy and awareness of the holobiont might shape an individual’s relationship with nature. One study found a significant association between attitudes towards microbes and both duration and frequency of visits to natural environments (Robinson et al. 2021). A higher frequency of weekly visits to nature and a longer duration spent in nature per visit were significantly associated with positive attitudes towards microbes. However, there are no studies on whether one’s perception of the holobiont concept influences one’s nature connectedness.

Recent work suggests that the microbiome—the collective genetic material of microorganisms within a given environment—may play a role in shaping human perceptions and behaviours (Dinan et al. 2015; Villa and Sánchez-Pérez 2021). Indeed, the microbiome is not just a biological phenomenon but a conceptual bridge between humans and the environment, as microbial communities link us to the air, soil, water, plants and other organisms we interact with daily (Elhaik et al. 2021; Ishaq et al. 2021; Robinson and Breed 2023). This interconnectedness aligns with frameworks such as One Health and Planetary Health, which emphasise the indivisibility of animal, environmental and microbial health (Banerjee and Heijden 2023). Despite the relevance of these perspectives, public awareness of the holobiont concept remains limited, and the potential psychological impacts of its perception—particularly regarding nature connectedness—have yet to be fully explored.

To investigate whether holobiont literacy influences nature connectedness, we conducted a randomised controlled study in which participants were exposed to either holobiont-related information or a neutral control condition. Participants were recruited globally through open recruitment on social media. The call for participation invited people to “Take a few moments to do our nature connectedness survey” and “Please fill in our 5–10 min survey on nature connection”. Upon clicking on the link, participants were then randomly assigned to either a holobiont treatment group, who watched an educational video and received supplementary text and image-based information about the holobiont, or a control group, who watched a neutral video, received text and image-based information on the importance of sleep and did not receive holobiont-related content. To measure nature connectedness, participants completed a validated nature connectedness scale (the Connectedness to Nature Scale; Mayer and Frantz 2004) before and after exposure to the assigned content. The study aimed to answer three key research questions:

- Does prior knowledge of the holobiont correlate with nature connectedness?
- Does exposure to holobiont information impact participants’ nature connectedness scores?
- Which components of the natural world (i.e. inanimate phenomena, animals, plants or microbes) do people feel more connected to?

By expanding our understanding of how microbial literacy intersects with nature connectedness, this research provides a novel framework for exploring human–nature relationships through the lens of the microbiome.

## Materials and methods

### Design and materials

The study, including the hypotheses, was preregistered on the Open Science Framework platform prior to seeking ethical approval and commencing data collection.^1^ The complete time-stamped pre registration, materials and aggregated dataset can also be accessed on the platform.^2^ The study employed a randomised controlled experimental design to investigate the relationship between holobiont literacy and nature connectedness. Participants were randomly assigned to one of two conditions: the holobiont (H) condition or the control (C) condition. It received ethical approval through the Division of Psychology, Sociology and Education committee at Queen Margaret University, Edinburgh. The study followed a pretest/post-test design, with all participants completing a baseline measure of nature connectedness before exposure to their assigned condition. Nature connectedness was assessed using the *Connectedness to Nature Scale* (CNS), a 14-item, 5-point Likert-scale instrument designed and validated to measure adults’ affective and experiential relationship with the natural world (Mayer and Frantz 2004). Higher scores on the CNS indicate a stronger sense of nature connectedness. Several questions are negatively framed (i.e. “I often feel disconnected from nature”, “When I think of my place on Earth, I consider myself to be a top member of a hierarchy that exists in nature” and “My personal welfare is independent of the welfare of the natural world”). These were reverse-coded prior to summing the scores, so that higher scores consistently indicate greater connectedness to nature. The internal reliability (Cronbach’s alpha) for the scale ranged from 0.86 to 0.95 (before and after the intervention), which is slightly higher than the reliability reported in previous studies (Mayer and Franz, *α* = 0.79–0.84), indicating strong measurement consistency.

### Procedure

Upon accessing the survey, participants were automatically allocated to either the holobiont or control condition using a custom Python function for randomisation. The system ensured a roughly equal number of participants in each group by using stratified randomisation. Once allocated, participants were directed to the corresponding survey. Following the randomised allocation, participants in the holobiont condition completed a microbiome knowledge questionnaire to assess their prior understanding of microbial ecology and the holobiont concept. The participants then watched a short educational video explaining the holobiont concept and read accompanying text and image-based materials that further elaborated on the role of symbiotic microbes in shaping human biology and ecology.^3^ In contrast, participants in the control condition watched a neutral video about the importance of rest and read associated materials that did not mention microbiomes or holobionts.^4^

After information exposure, participants in both conditions retook the CNS questionnaire to assess potential changes in nature connectedness and provided demographic information (age, gender and location). To control for potential confounding factors, participants in the control condition also completed the microbiome knowledge questionnaire at this stage, thereby allowing assessment of their baseline understanding of the holobiont concept.

A qualitative component was included for participants in the holobiont condition, asking them to reflect on how learning about the holobiont concept influenced their perception of nature. These open-ended responses were analysed to identify emergent themes, providing additional insight into the potential psychological effects of holobiont literacy. At the conclusion of the study, participants received a debriefing note explaining the study’s purpose and were given the opportunity to view the alternative video if they wished. This ensured transparency and allowed participants to engage with both perspectives presented in the experiment.

**Fig. 1:**
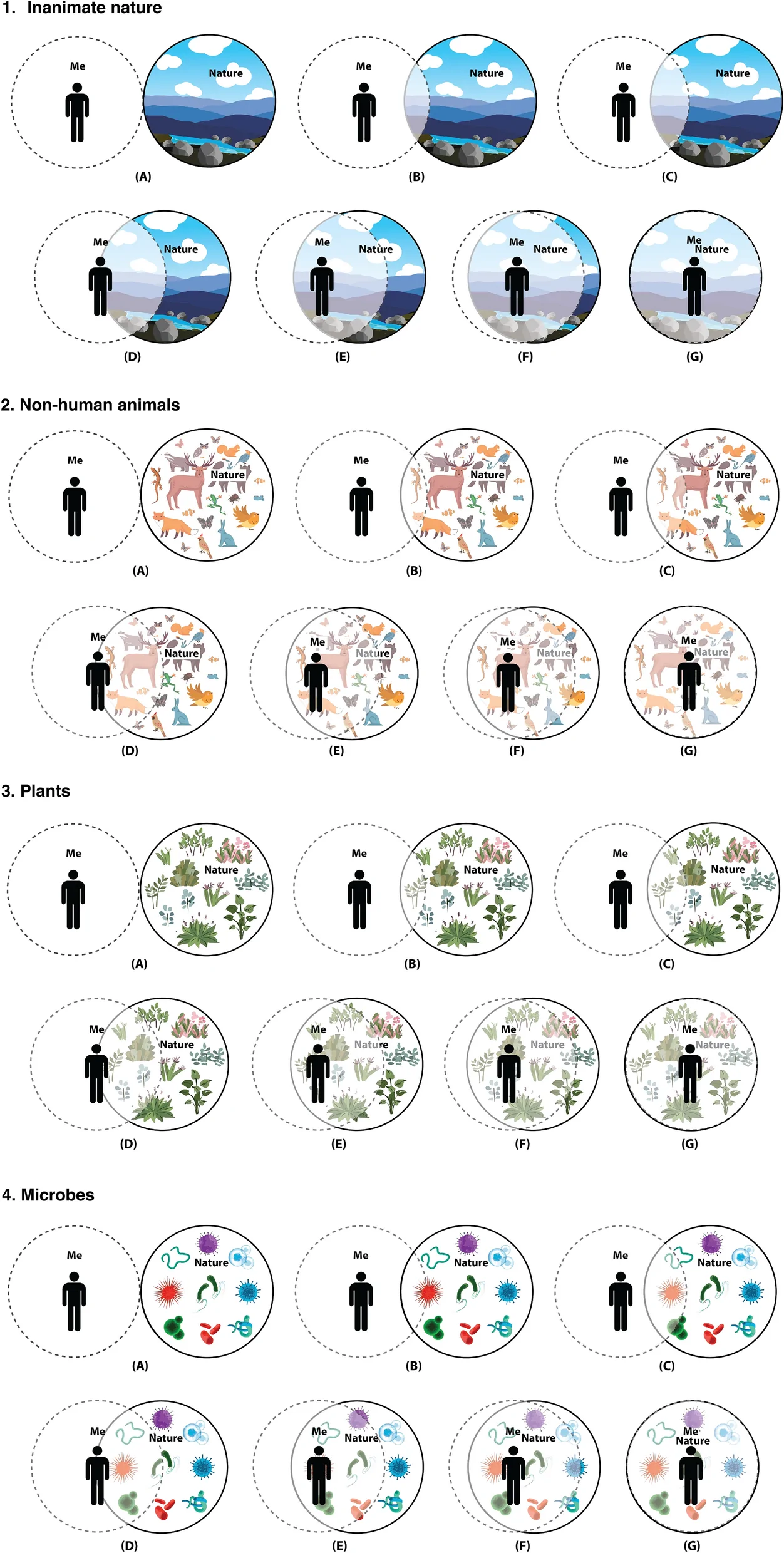
The modified Illustrated Inclusion of Nature in Self Scale. (1) Inanimate nature, (2) non-human animals, (3) plants and (4) microbes

To explore whether people felt more strongly connected to inanimate phenomena, animals or plants than microbes, we used a modified version of the Illustrated Inclusion of Nature in Self Scale (IINS; Fig. 1) (Schultz 2002; Kleespies et al. 2021). For this survey, participants were asked: “*Looking at the figure above, choose the appropriate letter (A–G) to indicate which of the pictures best represents your relationship to [inanimate nature], [plants], [other non-human animals], [microbes]”.* The participants were asked before and after the intervention.

### Participants

An a priori power analysis was conducted to determine the minimum number of participants required to detect an effect. It was conducted using G*Power version 3.1.9.7 (Faul et al. 2007). Results indicated that the required sample size to achieve 80% power to detect an effect of *d* = 0.4 (Cohen 1988) at a significance level of *α* = 0.05 was *n* = 82 per group for a one-tailed Mann–Whitney test. Thus, the obtained sample size of *n* = 91 for the treatment group and *n* = 98 for the control group is adequate to test the main study hypothesis.

### Data analysis

All quantitative data analysis was done in Python (Version 3.11.8; Python Software Foundation 2025) and Visual Studio Code (Version 1.97; Microsoft Corporation 2025). Shapiro–Wilk tests were conducted to assess the normality of nature connectedness scores across all conditions. Given the significant deviations from normality (*p* < 0.05), nonparametric tests were used for subsequent analyses. To address missing data, we used multiple imputation via the iterative imputer function from the sklearn.impute module in Python. This method estimates plausible values based on observed data, minimising bias and preserving statistical power. To determine whether there were baseline differences in nature connectedness between the two groups, a Mann–Whitney U test was performed on the pre-intervention scores. Wilcoxon signed-rank tests were conducted to assess whether nature connectedness scores changed within each group following the intervention. To test the effects of prior knowledge of the holobiont on nature connectedness, we coded correct answers with “1” and incorrect or “I don’t know” answers with “0”, resulting in a minimum holobiont knowledge score of 0 and a maximum score of 7. We then applied a linear regression model. Since the residuals were not normally distributed, a robust linear model (RLM) was used instead of ordinary least squares (OLS) regression. We also used this model to account for age, gender and geographic region on nature connectedness. RLM is more resistant to outliers and violations of normality assumptions, ensuring a more reliable estimation of the relationship between prior knowledge and nature connectedness (NC) score. Mann–Whitney U tests were conducted to compare nature connectedness scores between the control and holobiont groups at both time points on the IINS. A Friedman test was conducted to assess differences between inanimate objects, animals, plants and microbes before and after the intervention. All visualisations were done in Python and Adobe Illustrator (Version 29.3.1; Adobe 2025).

## Results

The average age of the participants was M_years_ = 48 (range 21–86; SD = 14), and the sex ratio was 132 female, 54 male and 4 “Other”. The participants came from Africa (*n* = 3), the United States (*n* = 9), Asia (*n* = 3), Europe (*n* = 143) and Oceania (*n* = 32). The mean CNS scores for the holobiont treatment group were M = 53.1 (SD = 7.5) before the intervention and M = 53.8 (SD = 8.7) after the intervention. The mean CNS scores for the control group were M = 52.3 (SD = 7.6) and M = 52.4 (SD = 9.5) after the intervention. The mean holobiont knowledge scores were M = 3.8 (SD = 2.6) for the holobiont treatment group and M = 3.3 (SD = 2.6) for the control group.

### The effect of prior knowledge of the holobiont on nature connectedness

**Fig. 2:**
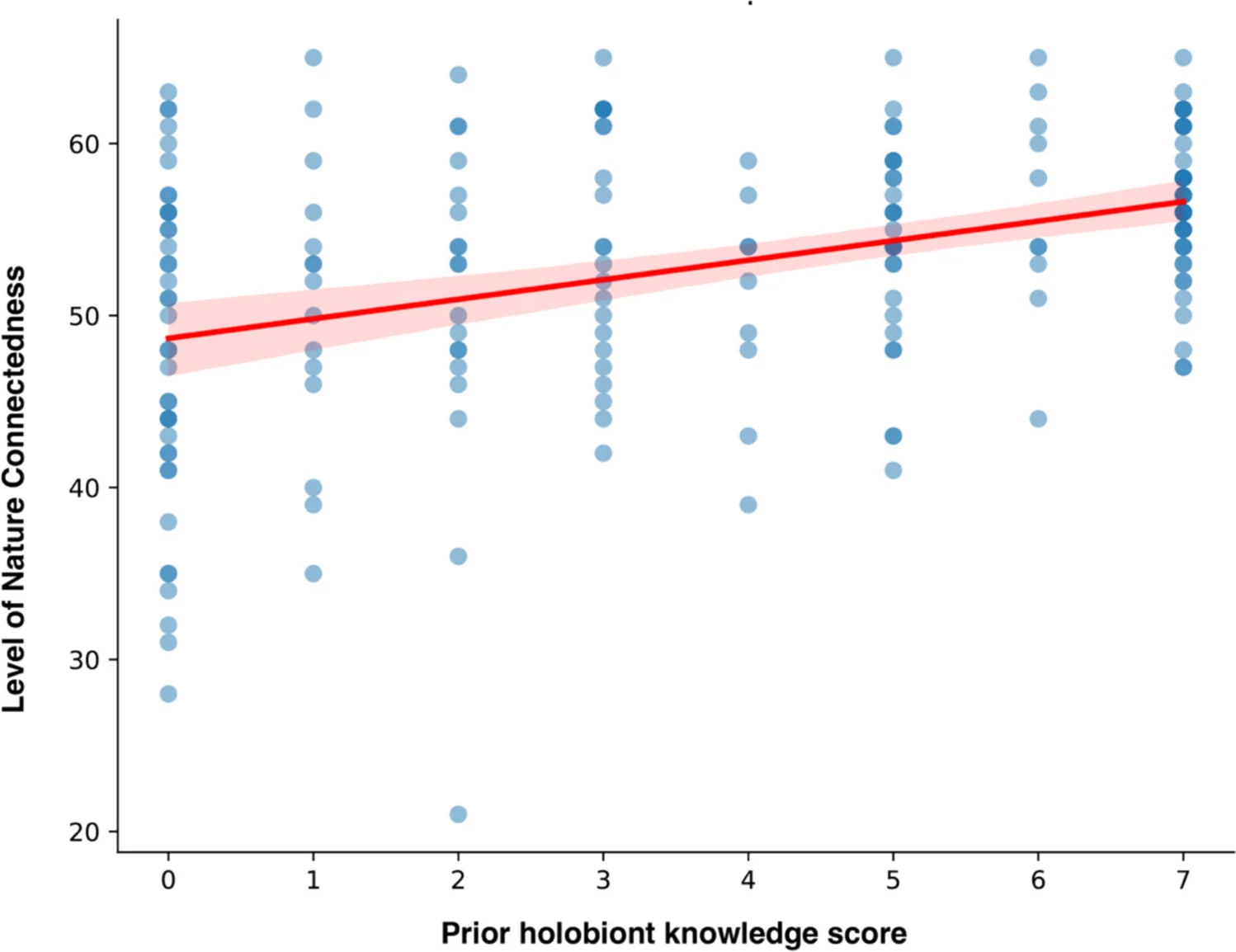
Regression scatter plot showing the relationship between prior holobiont knowledge and nature connectedness

The regression model revealed a significant positive association between prior knowledge and NC score (Fig. 2). The model intercept was 49.44, indicating that individuals with no prior knowledge of the holobiont had a baseline NC score of approximately 49.4 (out of 70). The regression coefficient for prior knowledge was 1.03 (*p* < 0.001), meaning that for every one-unit increase in prior knowledge, the NC score increased by approximately 1.03 points. This suggests that greater awareness of the holobiont concept is associated with stronger feelings of nature connectedness. The 95% confidence interval for the prior knowledge coefficient ranged from 0.64 to 1.42, reinforcing the robustness of the observed effect. A total of 188 participants were included in the analysis (two were removed due to insufficient data coverage), and the model converged after 13 iterations, which is typical for robust regression.

In terms of demographic factors, gender had a significant impact on nature connectedness. Men (*β* = − 2.54, SE = 1.11, *p* = 0.020) scored significantly lower on nature connectedness than women, indicating that male participants had a weaker sense of connection to nature (f^2^ = 0.032, 95% CI [− 4.72, − 0.36]). However, for individuals identifying as non-binary or other genders (*β* =  − 1.32, *p* = 0.840), the difference was not statistically significant. Age did not significantly influence nature connectedness (*β* = 0.01, *p* = 0.760), suggesting that nature connectedness was relatively stable across age groups in this sample. Similarly, geographic region did not show any significant effect on nature connectedness, as all regional coefficients were non-significant (all *p* > 0.050).

### The effect of holobiont information on nature connectedness

#### Within-group comparisons (pre- vs. post-intervention)

**Fig. 3:**
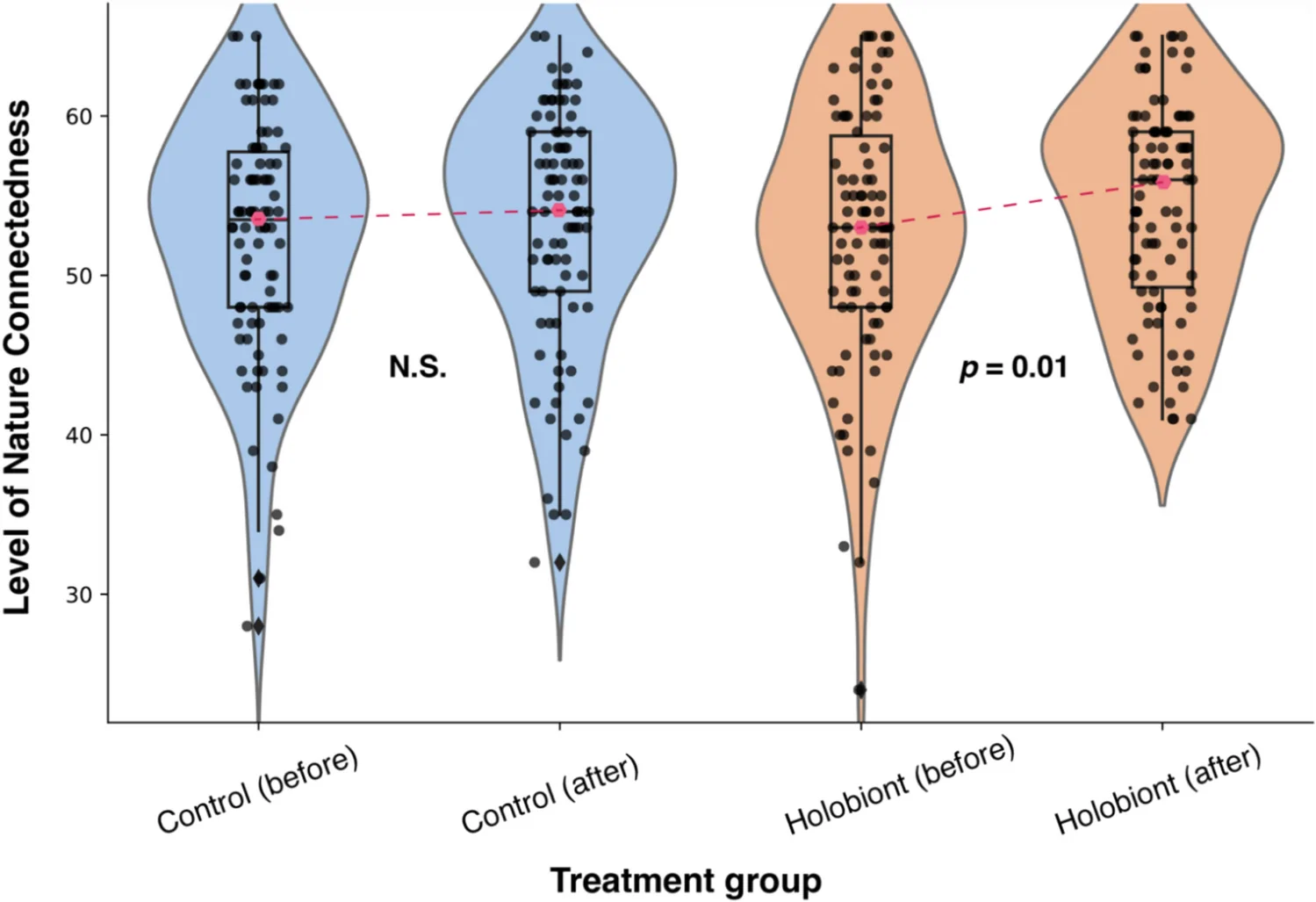
Violin boxplots of results from the connectedness to nature scale survey. Red dash, mean trend line; NS, not significant

Participants in the holobiont group showed a statistically significant increase in nature connectedness following exposure to holobiont-related materials (*W* = 1033.5, *p* = 0.01), suggesting that learning about the holobiont concept may positively influence participants’ perceived connection to nature (Fig. 3). The effect size of the holobiont intervention was moderate (*r* = 0.304, 95% CI [0.150, 0.364]). In the control group, there was no significant change in scores between the pre- and post-intervention assessments (*W* = 1112, *r* = 0.128, 95% CI [0.034, 0.339], *p* = 0.44), indicating that watching a neutral video did not affect nature connectedness.

#### Comparison between the control and treatment groups

The results revealed no significant difference between the control (before) and holobiont (before) groups (*U* = 3726.5, Cliff’s delta = − 0.080, 95% CI [− 0.216, 0.113], *p* = 0.355) or the control (after) and holobiont (after) groups *U* = 3545, Cliff’s delta = − 0.125, 95% CI [− 0.268, 0.060], *p* = 0.148), suggesting comparable starting and post-intervention points.

### Which components of the natural world (i.e. inanimate phenomena, animals, plants or microbes) do people feel more connected to?

#### Differences between control and holobiont groups

**Fig. 4:**
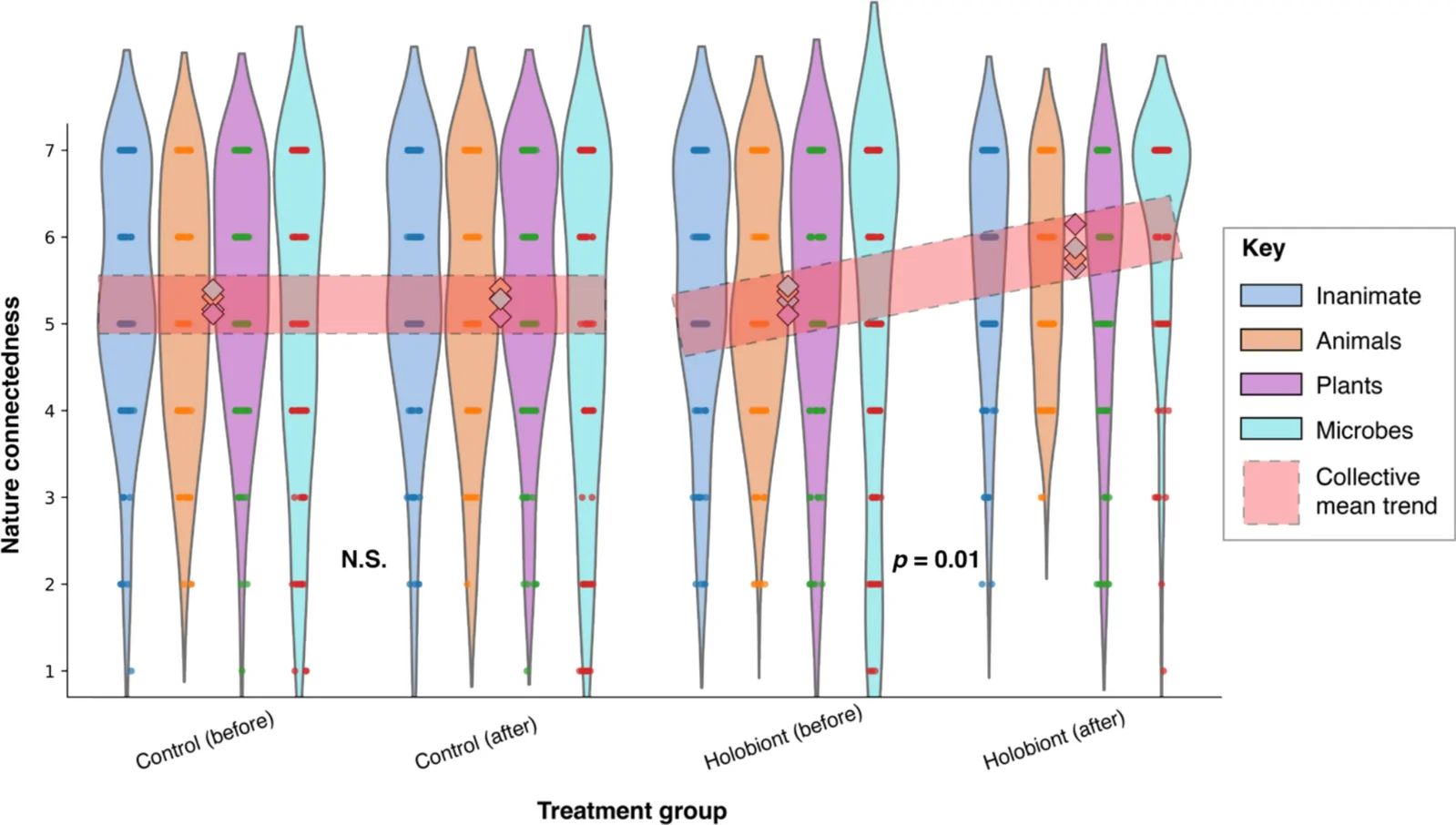
Violin boxplots of the Illustrated Inclusion of Nature in Self Scale results for each component and treatment (before and after the information intervention)

Before the intervention, there were no significant differences between the control and holobiont groups in connectedness to inanimate nature (0.463, 95% CI [0.380, 0.547], *p* = 0.376), (non-human) animals (0.478, 95% CI [0.398, 0.561], *p* = 0.595), plants (0.510, 95% CI [0.426, 0.593], *p* = 0.815) or microbes (0.485, 95% CI [0.402, 0.568], *p* = 0.718). After the intervention, there were no significant changes in nature connectedness over time for the control group (all *p* > 0.050), indicating that participants who did not receive the holobiont intervention maintained stable perspectives on nature connectedness. In contrast, the holobiont group showed significant increases in connectedness to inanimate nature (0.348, 95% CI [0.139, 0.557], *p* = 0.001), animals (0.399, 95% CI [0.190, 0.608], *p* < 0.001), plants (0.328, 95% CI [0.119, 0.537], *p* = 0.002) and microbes (0.573, 95% CI [0.365, 0.782], *p* < 0.001), suggesting that exposure to holobiont information strengthened their sense of connection across multiple aspects of nature (Fig. 4).

### Qualitative results

The results of the qualitative component show that participants viewed the holobiont information video overwhelmingly positively. Some people had never thought of themselves as a community of life, and to others, it brought a new perspective and realisation of how dependent we are on our microbiomes. However, there was one negative component—relating to “guilt” of getting a C-section (Table 1).

**Table 1:** Summary of qualitative responses to the holobiont information video (incl. a word cloud of common terms)

Remark	Emotion
“A sense of connection to my genetic and historical context. I thought on all the experiences that might have had an effect on my microbiome”.	Positive
“I loved it…powerful video… thought-provoking…shows that we know so little about our relationship with living/non-living entities beyond a limited understanding of "self"…. I've long had a habit of referring to myself in plural… me, myself, and I….now I know it is far more complex!”	Positive
“If I have a baby (which seems unlikely) I’d like to have a c-section, and it made me feel guilty”.	Negative
“I felt smug about having never tried to erect too strong a boundary between me and natural world and always having trusted my gut(!) instinct on what safe”.	Positive
“While I feel very connected to nature and knew that humans are hosts to bacteria, viruses etc., I did not know the terminology "holobiont". Watching the information made me feel even more a part of nature. I sometimes feel like humans are such a drain on ecosystems and need to remember we are a part of it in very positive ways as well. I do know this, but it is sometimes difficult to remember how amazing even just the human body is. I have been thinking about the next steps of life and having a child and the video sparked some of my own fears about childbirth. Not because I am scared of childbirth, but I am worried I will be forced into having a c-section. I have done a decent amount of reading in this area which is probably why I knew about the benefits of a vaginal birth related to the video's contents”.	Positive and negative
“Amazing!! Exceptional science communication—took an abstract concept and made it feel dynamic, emotional, and connected to daily life. Definitely inspired me to want to learn more about and tend to my own microbiome”.	Positive
“I felt whole, fulfilled, learning that I am a community of organisms as I am an organism in an ecosystem. Wow, I never thought of myself as a community”.	Positive
“It was very informative thank you as I had Nfi about that stuff”.	Positive
“It gives a different perspective to myself and my body and made me realise how dependent we are on our microbiome which is generally overlooked because they are invisible to our eyes”.	Positive

## Discussion

This study demonstrates that holobiont literacy may play a significant role in shaping nature connectedness. Our results indicate that individuals with greater prior knowledge of the holobiont concept reported higher levels of nature connectedness. Moreover, exposure to holobiont-related information enhanced these perceptions. These findings provide novel insights into how microbiome literacy influences psychological relationships with the natural world and extend existing theories on nature connectedness by incorporating a microbial dimension.

The significant association between prior knowledge of the holobiont and nature connectedness suggests that individuals who already conceptualise themselves as interconnected biological collectives exhibit a stronger emotional and cognitive bond with nature. This aligns with existing frameworks that suggest nature connectedness is influenced by cognitive awareness of ecological relationships (Andrews 2018; Mundaca et al. 2021). Our study builds on this by demonstrating that microbial awareness may serve as an additional, previously underexplored, cognitive mechanism for enhancing nature connectedness. However, it could also be that people with higher nature connectedness are more likely to seek out information on microbiomes and the holobiont concept. For instance, feeling more connected to nature may elicit heightened curiosity to explore multiple dimensions of biodiversity (including the invisible).

### Holobiont literacy as a psychological and educational tool

Exposure to holobiont information resulted in a significant increase in nature connectedness scores among participants, with a moderate effect size, indicating a meaningful change in perceived connection to nature. The effect size in our intervention is similar to what a meta-analysis of interventions that use direct nature exposure activities found (Sheffield et al. 2022). This suggests that framing humans as holobionts—rather than as isolated individuals—may strengthen psychological and emotional ties to the natural world. Given that the microbiome serves as an interface between humans and their environment, educating the public about the holobiont concept could be a valuable tool for promoting pro-environmental attitudes and behaviours. Previous studies have demonstrated that nature connectedness is malleable and can be enhanced through directexposure to natural environments, educational interventions and mindfulness-based activities (Richardson et al. 2020; Barrable et al. 2021). Our findings suggest that microbiome education may provide another pathway to increasing nature connectedness, reinforcing the reality that humans are biologically embedded within broader ecological systems. Future interventions could explore how incorporating holobiont literacy into environmental education may influence conservation attitudes and ecological stewardship. Moreover, further work on how types of educational material affect the extent of change in individuals’ nature connectedness would be of interest. Finally, examining more diverse participant groups, including different ages and cultures, could further inform how to incorporate holobiont literacy into environmental education or well-being programmes.

### Nature connectedness and components of the natural world

An unexpected finding was that, before the intervention, participants did not exhibit significant differences in connectedness across different components of nature (inanimate, animals, plants and microbes). This was unexpected, given evidence that human–nature relationships are shaped by familiarity, visibility and affective responses, and that microbes, in particular, are often perceived negatively or overlooked due to their invisibility (Schultz 2002; Robinson et al. 2021). However, after exposure to holobiont-related information, the holobiont group reported significantly increased connectedness to all categories, including inanimate nature and microbes. This broad effect suggests that holobiont literacy encourages a more holistic view of ecological interconnectedness, rather than solely influencing attitudes towards microbial life. One possible explanation is that understanding the holobiont concept encourages a systems thinking approach to nature. By recognising that human health and well-being depend on symbiotic microbial communities, individuals may become more attuned to the intricate relationships within natural systems. This supports previous research suggesting that ecological literacy is positively associated with nature connectedness and environmental responsibility (Otto and Pensini 2017; Jia and Wang 2024).

### Implications for conservation psychology and environmental education

The results of this study hold important implications for conservation psychology and environmental education. Given that nature connectedness is linked to pro-environmental behaviour (Teixiera et al. 2023), integrating holobiont literacy into public outreach and education programmes could be an effective strategy for enhancing ecological attitudes and behaviours. Programmes that frame microbial life as an essential part of human identity and environmental health may help shift perceptions away from anthropocentric and germophobic narratives towards a more interconnected, mutualistically symbiotic worldview (Boyd et al. 2024).

Furthermore, the significant effect of holobiont literacy on increasing nature connectedness suggests that microbiome science should be better integrated into sustainability education curricula. Educators could leverage concepts such as symbiosis (Robinson and Barrable 2023), microbial ecology and the human microbiome to illustrate the interconnectedness of life and reinforce the psychological and ethical imperative for biodiversity conservation and restoration.

The qualitative component revealed that engaging in science communication is important in shaping people’s views. From a neuroscience perspective, the way information is framed and delivered can significantly influence attention, emotional engagement and memory retention (Powell et al. 2019; Carfora et al. 2021). The salience network—a brain system that helps filter relevant stimuli and prioritise important information—plays a crucial role in determining whether individuals engage with and internalise scientific concepts (Seeley 2019). When new information, such as the holobiont concept, is framed in a way that resonates with personal identity and lived experience, it is more likely to activate the salience network, leading to deeper cognitive processing and stronger retention.

One respondent reported feeling uncomfortable when learning about the role of microbes in childbirth, particularly regarding C-sections and their potential impact on infant microbiome development. This highlights an important consideration in science communication: while factual information is crucial, its presentation can trigger affective responses, particularly when it concerns deeply personal topics such as birth, health and parenting. Science communication that effectively highlights the interconnectedness of human and microbial life may influence the default mode network (DMN), a system involved in self-referential thinking (Knyasev et al. 2021). By integrating the holobiont concept into narratives that emphasise our embeddedness within ecosystems, we may facilitate shifts in perception from anthropocentric worldviews towards a more ecocentric perspective. This shift is important for promoting pro-environmental attitudes and behaviours and for improving public understanding of biodiversity and its role in planetary and human health. Moreover, research suggests that engaging, multimodal communication—such as storytelling, visual imagery and interactive digital media—can enhance learning by leveraging the brain’s reward pathways (Dubinski and Hamid 2024). This suggests that to improve public literacy on the holobiont and biodiversity, communication strategies should focus on making abstract microbial concepts tangible, emotionally salient and personally relevant––while inspiring curiosity rather than fear. By doing so, we may enhance awareness and promote a deeper sense of connection to the ecosystems that sustain us.

### Limitations and future directions

Despite the strengths of this study, several limitations should be acknowledged. First, the sample was self-selected and may have been biased towards individuals with pre-existing interest in nature and microbiology, given the recruitment process. Future studies should aim for more diverse and representative samples to enhance generalisability. Second, the intervention in this study consisted of a short video and accompanying text-based materials. While the findings suggest that even brief exposure to holobiont-related content can influence nature connectedness, future studies should explore the effects of more immersive and interactive educational experiences, such as virtual reality simulations or field-based microbiome exploration activities. Finally, longitudinal studies are needed to assess whether the observed effects of holobiont literacy on nature connectedness persist over time. If microbial awareness can produce lasting changes in how individuals perceive and interact with nature, this could have profound implications for conservation psychology and public health.

An important implication of these findings is the potential value of introducing holobiont and microbiome concepts early in life. Developing an understanding of humans as multispecies assemblages from a young age may help encourage a more integrated sense of self within nature, supporting the development of ecological awareness and pro-environmental attitudes. Early-life microbiome literacy could therefore help shape more sustainable worldviews, aligning with broader One Health and planetary health frameworks. Embedding these concepts within educational curricula may contribute to long-term shifts in how individuals perceive their relationship with the natural world, with potential implications for both human well-being and environmental sustainability.

## Conclusion

This study provides the first empirical evidence that holobiont literacy can significantly influence nature connectedness, with an effect size similar to that found in direct exposure nature connectedness interventions. Individuals with greater prior knowledge of the holobiont concept reported stronger connections to nature, and exposure to holobiont-related information further enhanced nature connectedness scores. Additionally, learning about the holobiont strengthened participants’ perceived connection to all components of the natural world, suggesting that microbiome awareness promotes a more holistic ecological perspective. These findings highlight the potential of microbiome education as a tool for strengthening human–nature relationships, with implications for environmental psychology, education and conservation initiatives. By integrating holobiont literacy into public discourse and educational curricula, we may cultivate a deeper appreciation for the interconnectedness of life and inspire more sustainable interactions with the natural world.

## Data Availability

Aggregated quantitative data and materials are available here https://osf.io/kgcd7/overview.

## Footnotes

1 https://osf.io/kgcd7/.

2 https://osf.io/q2n5v/overview.

3 https://www.youtube.com/watch?v=5DTrENdWvvM.

4 https://www.youtube.com/watch?v=FEmSM5pWk9E.
